# The Lunacy Blue Book

**Published:** 1893-10-14

**Authors:** 


### THE LUNACY BLUE BOOK.

The forty seventh annual report of the Commissioners in Lunacy discloses the unpleasant fact that when the year 1892 ended there were known to be 1,974 more insane persons in England and Wales than there were on January 1st, the total number of the registered insane being now 89,822. Of this total 80,893 are paupers in the Lunacy Act acceptation of the term, that is, they are maintained wholly or in part by some union, county, or borough, and not fewer than 1,440 of these paupers are kept in private asylums at a cost very far in excess of what it would be in public asylums. The ratepayers are thus subjected to an increase of taxation easily avoidable. It is a mystery why some enterprising member of the County Council does not take this matter up and expose the system which permits it, a system which we have already commented on in these columns. As the population of the country increases it must be looked for that the gross number of lunatics will increase also; and not only do we find this, but we learn that the proportion of the insane to the sane increases. In the year 1869 the ratio was 23·93 per ten thousand, while in 1893 it had gone up to 30·21. It must not be assumed that the actual increase of insanity among us runs parallel with the increase of insane persons in asylums. As the Commissioners point out, there is a larger proportion of incurables among the admissions now than formerly, and, moreover, the public have learnt to place confidence in the management of public asylums, and, as a consequence, many patients above the rank of paupers are admitted. The friends of these patients reimburse the guardians for the cost of maintenance, and as many of these cases turn out to be incurables, they swell the increment of our registered insane. While the number of pauper patients has considerably, even greatly, increased during the year, it is curious to note that the private patients have decreased by 73. The general recovery rate is a fraction under 40 per centum, and the death-rate a fraction under 10 per centum, the former being calculated on the admissions of the year, and the latter on the average number resident during the year. The recovery rate and the death rate are both a trifle lower than they were in 1891; and we have here an explanation of part of the increase in the total number of insane persons under treatment. Table XI. shows the distribution of pauper lunatics in the various counties as regards residence in asylums, workhouses, and private houses, and we notice some strange discrepancies. For instance, the County of London has a total of 17,214 pauper lunatics. Of this total, 62·7 per cent. are in asylums, 35·8 per cent. in workhouses, and only 1·5 per cent. in private houses. On the other hand, Cardigan has a total of 305, and we find 49·5 per cent. in asylums, 11·5 per cent. in workhouses, and 39 per cent. in private houses. It would seem that the County of London had an increase of 447 pauper lunatics during the year, and assuming this increase to be fairly constant it will be necessary to build an asylum for one thousand patients every two years to keep pace with this ever-swelling wave of pauper lunacy. Ways of stemming this torrent, or at least of abating it, have been pointed out again and again, but they have fallen on deaf ears. Indeed, one of the most prominent facts, alike instructive and deplorable, is the cry raised for further accommodation in four-fifths of our English asylums. A new asylum is being built for Somerset for 450 patients, at a cost of £85,000, although it has been proved that a good asylum may be built for a much smaller sum; and another for Sunderland, at £61,500. The County of Stafford, the Isle of Wight, the Borough of Blackburn are taking steps to build. Old asylums are being added to; in some cases at great expense. Durham is spending £11,000 on a laundry. A block for 252 patients at Glamorgan is to cost £43,000. At Banstead £11,900 is to be spent on infirmary wards. At Wandsworth, a block for 200 idiots is to cost the nice little sum of £33,000. At Menstone, a block for 600 patients is estimated at nearly £86,000, with certain adjuncts. Newcastle is to add 311 beds, but the estimate is not given. Nor is this all. The Three Counties' Asylum, the Cheshire asylums, the asylums at Denbigh, Bodmin, Derby, and Devon, must all soon follow suit and extent their boundaries. The county and borough asylums contained 71,878 patients, and it is certain that a very considerable proportion of these would be, at one time or another, suicidally disposed. Notwithstanding this, only fourteen patients succeeded in committing the act during the year. Thirty years since, with a total of 25,799, there were twelve suicides in our public asylums, so that the proportion of sucides to patients is not much greater than one-third of what it was a generation since. Indeed, it is not probable that the next generation will see much diminution in the number of suicides in asylums. We have probably reached the low-water mark compatible with the proper and necessary freedom of the patients, and the practice of the present day is all tending to greater freedom and more trust. The average weekly cost of maintenance in county asylums is 8s. 11⅝d., and in borough asylums 10s.

